# Clinical epidemiology of infectious disease among patients with chronic kidney disease

**DOI:** 10.1007/s10157-018-1641-8

**Published:** 2018-09-03

**Authors:** Junichi Ishigami, Kunihiro Matsushita

**Affiliations:** Department of Epidemiology, Welch Center for Prevention, Epidemiology, and Clinical Research, Johns Hopkins Bloomberg School of Public Health, 2024 E. Monument St., Suite 2-600, Baltimore, MD 21287 USA

**Keywords:** Chronic kidney disease, Infections, Pneumonia, Bloodstream infections, Renal failure, Influenza vaccination, Pneumococcal vaccination

## Abstract

Infectious disease is recognized as an important complication among patients with end-stage renal disease, contributing to excess morbidity and health care costs. However, recent epidemiological studies have revealed that even mild to moderate stages of chronic kidney disease (CKD) substantially increase risk of infection. Regarding underlying mechanisms, evidence suggests various aspects of altered immune response in patients with CKD including impaired function of T cells, B cells and neutrophil. Multiple conditions surrounding CKD, such as older age, diabetes, and cardiovascular disease are important contributors in the increased susceptibility to infection in this population. In addition, several mechanisms impairing immune function have been hypothesized including accumulated uremic toxins, increased oxidative stress, endothelial dysfunction, low-grade inflammation, and mineral and bone disorders. In terms of prevention strategies, influenza and pneumococcal vaccines are most feasible and important. Nevertheless, the extent of vaccine utilization in CKD has not been well documented. In addition, antibody response to vaccination may be reduced in CKD patients, and thus a vaccine delivery strategy (e.g., dose and frequency) may need to be optimized among patients with CKD. Through this review, we demonstrate that infection is a major but underrecognized complication of CKD. As CKD is recognized as a serious public health issue, dedicated research is needed to better characterize the burden of infectious disease associated with CKD, understand the pathophysiology of infection in patients with CKD, and develop effective strategies to prevent infection and its sequela in this high risk population.

## Introduction

Chronic kidney disease (CKD) is a serious public health issue, affecting 8–16% of adult population worldwide [1]. Although historically cardiovascular disease has been considered as one of the most important CKD complications [2], an accumulating body of evidence has revealed that CKD is also an important risk factor for non-cardiovascular outcomes (e.g., cognitive decline [3], fracture [4], bleeding [5]). In this context, infection is probably the most important non-cardiovascular outcome since it poses the second leading cause of hospitalization after cardiovascular disease [2]. While it is well-recognized that infection risk is extremely high among patients with end-stage renal disease (ESRD), a few recent studies suggest that even less severe CKD substantially increases the risk of infection. Nevertheless, data on the epidemiology of infectious disease are still sparse in the entire CKD population including its mild to moderate stages. Such a knowledge gap is critical since the vast majority of CKD patients are at mild to moderate stages [2]. In this review, we will discuss the current evidence regarding the epidemiology of infectious disease in CKD. In the first section, we will discuss the incidence of infectious disease associated with CKD in inpatient and outpatient settings. In the second section, we will discuss possible mechanisms and contributing factors to the increased susceptibility to infection in CKD. In the third section, we will discuss infection prevention strategies primarily focusing on vaccination programs. We will also list some potential future directions in this context.

## Incidence of infectious disease associated with CKD

According to a report from the 2017 United States Renal Data System [6], the incidence of hospitalization was 614 per 1000 person-years in individuals aged 65 years or older with any stage of CKD, which was nearly 3 times higher as compared to the incidence of 214 per 1000 person-years in those without CKD. Regarding cause of hospitalization, cardiovascular disease was the leading cause of hospitalization, accounting for 23% of all-cause hospitalization. Infection was the second major cause, accounting for 21% of all hospitalizations—a burden almost identical to cardiovascular disease [6]. Thus, it is important to recognize infection as a leading cause of hospitalization among individuals with CKD.

Table 1 summarizes the characteristics of representative cohort studies investigating the association between eGFR and risk of infection. Regarding the risk of hospitalization with infection, previous studies consistently showed an association between lower eGFR and risk of hospitalization with infection [7–10]. The risk is substantially increased even at mildly to moderately reduced eGFR: as compared to those with eGFR ≥ 60 ml/min/1.73 m^2^, individuals with eGFR 30–59 ml/min/1.73 m^2^ had an approximately 50% higher risk of hospitalization with infection. This pattern was observed for all-cause infection, as well as type-specific infections. Although the low prevalence of eGFR < 30 ml/min/1.73 m^2^ in the general population tends to limit the statistical power, the risk is exponentially increased in eGFR < 30 ml/min/1.73 m^2^, with a 2–3 times higher risk compared to eGFR ≥ 60 ml/min/1.73 m^2^. The association between low eGFR and risk of infection tended to be stronger among younger adults than older adults [8]. This could be explained by the lower incidence rate of infection in younger adults, resulting in a substantial increase in the relative risk even with a modest increase in the absolute risk. In addition, younger adults with reduced eGFR might be likely to have a unique etiology of kidney disease such as glomerulonephritis, polycystic kidney disease, or severe diabetes (e.g., type 1 diabetes), posing a particularly high risk of infection.

**Table 1:** Characteristics of representative cohort studies assessing risk of infection across eGFR

eGFR	Year	Setting	High risk population*	Sample size	Mean age, years	Infection type	Crude IR per 1000 p-years	Relative risk (95%CI) by eGFR category (ml/min/1.73 m^2^)					
								≥ 90	60–89	45–59	30–44	15–29	< 15
Hospitalization with infection													
James	2008	Canada	Yes	25,675	75	Bloodstream infections	10.4	Ref		1.24 (1.01–1.52)	1.59 (1.24–2.04)	3.54 (2.69–4.69)	
James	2009	Canada	No	252,516	range, 18–54	Pneumonia	1.7	Ref		3.23 (2.40–4.36)	9.67 (6.36–14.69)	15.04 (9.64–23.47)	
James	2009	Canada	Yes	252,516	range, ≥ 75	Pneumonia	31.7	Ref		0.95 (0.85–1.05)	1.03 (0.92–1.16)	1.79 (1.55–2.06)	
Dalrymple	2012	US	Yes	5,142	72	All-cause	34.7	Ref	1.16 (1.02–1.32)	1.37 (1.14–1.66)	1.64 (1.28–2.12)		Excluded
Ishigami	2016	US	No	9,697	63	All-cause	23.6	Ref	1.07 (0.98–1.16)	1.48 (1.28–1.71)		2.55 (1.43–4.55)	Excluded
Outpatient and inpatient infections													
McDonald	2014	UK	Yes	191,709	71	LRTI	155.8	Ref		1.03 (1.01–1.04)	1.08 (1.05–1.10)	1.17 (1.13–1.22)	1.47 (1.34–1.62)
Xu	2017	Sweden	No	1,139,470	52	All-cause	95.0	Ref		1.08 (1.01–1.14)		1.53 (1.39–1.69)	
Infection-related death													
James	2009	Canada	No	252,516	range, 18–64	Pneumonia	0.3	Ref		2.54 (1.40–4.60)	13.15 (7.04-424.56)	23.35 (11.52–47.32)	
James	2009	Canada	Yes	252,516	range, ≥ 75	Pneumonia	4.9	Ref		1.22 (1.01–1.49)	2.03 (1.64–2.50)	4.94 (3.94–6.19)	
Wang	2011	US	No	7,400	61	All-cause	1.9	Ref		1.36 (0.81–2.30)	2.36 (1.04–5.38)		Excluded
Ishigami	2016	US	No	9,697	63	All-cause	4.1	Ref	0.99 (0.80–1.21)	1.62 (1.20–2.19)		3.76 (1.48–9.58)	Excluded

*The study investigated population at high risk of infection (e.g., older adults, diabetes)

*IR* incidence rate, *eGFR* estimated glomerular filtration rate, *LRTI* lower respiratory tract infections

Increased risk of infection associated with reduced eGFR was also observed in ambulatory settings (Table 1) [11, 12]. Of note, although the association (i.e., relative risk) for outpatient infections seems weaker compared to inpatient infections, outpatient infections are much more common than inpatient infections. The incidence rates including outpatient infections ranged 100–150 cases per 1000 person-years, which was 3–5 times more frequent as compared to the incidence of hospitalization with infection [7–10]. Thus, although outpatient infections should have less prognostic impact than inpatient infections, they still pose a significant burden on CKD patients in terms of excess clinic visits and frequent antibiotic prescriptions, which reduce patients’ quality of life, impact health care costs, and induce multidrug resistant microorganisms [13, 14].

Previous studies also reported an increased risk of infection-related death associated with reduced eGFR (Table 1) [8, 15, 16]. However, we should interpret those results carefully since definitions of infection-related death varied across studies. For example, in a study of 38,520 individuals with eGFR < 60 ml/min/1.73 m^2^ using data from the electronic medical record-base registry in Ohio, the leading causes of death were cardiovascular disease (34.7%) and malignant neoplasms (31.8%), and deaths due to infections only accounted for 1.7% (influenza and pneumonia), and 1.4% (septicemia), respectively [17]. However, in a secondary analysis of the Trial to Reduce Cardiovascular Events With Aranesp Therapy [18], cause of death was centrally adjudicated, and infection was the second leading cause of death after cardiovascular death accounting for ~ 35% of all-cause mortality.

**Fig. 1:**
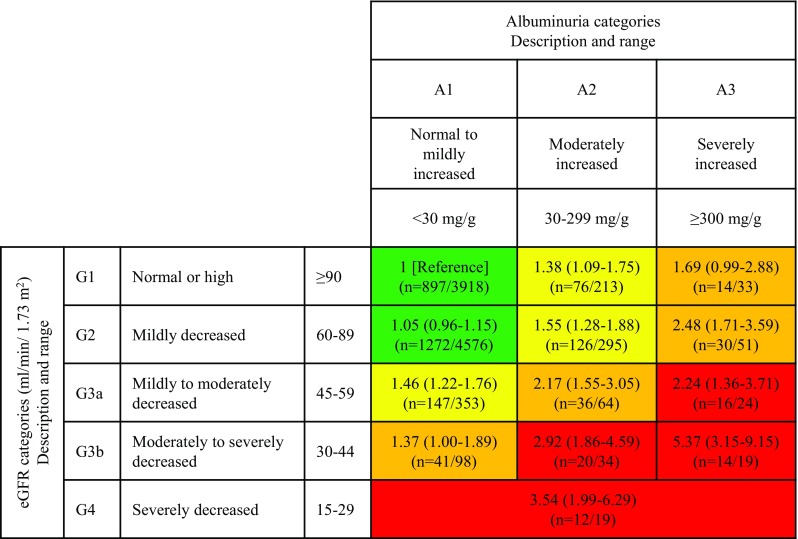
Adjusted hazard ratio of hospitalization with infection by eGFR and ACR categories. *GFR* glomerular filtration rate, *ACR* albumin-to-creatinine ratio. Green: low risk; yellow: moderately increased risk; orange: high risk; red, very high risk. For each category, hazard ratio and its 95% confidence interval were presented in the first row, and *n* = denotes number of events and number of individuals in the second row. The model was adjusted for age, race, sex, body mass index, smoking status, alcohol consumption, education level, use of antineoplastic agents and steroids, hypertension, diabetes, history of cancer, chronic obstructive pulmonary disease, prior heart failure, prior coronary disease, and prior stroke. Reprinted from reference 15 with permission

As compared to eGFR, fewer studies have examined albuminuria (Table 2). Among patients aged 65 years or older with diabetes, persons with positive dipstick proteinuria had nearly 10% higher risk for lower respiratory tract infections and nearly 30% higher risk for pneumonia or sepsis compared to those without [11]. In the Atherosclerosis Risk in Communities study, we observed a strong dose–response association between urinary albumin-to-creatinine ratio (ACR) and risk of hospitalization with infection, and this association was independent of eGFR (Table 2) [15]. Indeed, when assessed in the context of CKD risk stage according to the Kidney Disease Improving Global Outcomes (KDIGO) [19], there were multiplicative contributions of low eGFR and high ACR to the risk of hospitalization with infection (Fig. 1): within each eGFR category, risk of hospitalization with infection was higher with higher ACR in a graded fashion. Importantly, those with preserved kidney function (i.e., eGFR ≥ 60 ml/min/1.73 m^2^), but ACR ≥ 300 mg/g had an equivalent or even greater infection risk compared to those with moderately to severely reduced kidney function, but without albuminuria. Thus, in addition to reduced eGFR, health care providers should recognize albuminuria as an important risk factor of infection.

**Table 2:** Characteristics of representative cohort studies assessing risk of infection ACR

ACR	Year	Setting	High risk population*	Sample size	Mean age, years	Infection type	Crude IR per 1000 p-years	Relative risk (95%CI) by ACR category (mg/dL)			
								< 10	10–29	30–299	300+
Hospitalization with infection											
Ishigami	2016	US	No	9,697	63	All-cause	23.6	Ref	1.34 (1.20–1.50)	1.56 (1.36–1.78)	2.30 (1.81–2.91)
Outpatient and inpatient infections											
McDonald	2014	UK	Yes	191,709	71	LRTI	155.8	Ref (Dipstick negative)		1.07 (95% CI, 1.05–1.09) (Dipstick positive)	

*The study investigated population at high risk of infection (e.g., older adults, diabetes)

*IR* incidence rate, *eGFR* estimated glomerular filtration rate, *LRTI* lower respiratory tract infections

## Pathophysiological mechanisms increasing infection risk in CKD

### Impaired immune system in CKD

Impaired immune system has been recognized in CKD patients. For example, in patients with reduced kidney function, the number of lymphocytes, primarily the B lymphocyte and CD4^+^ T lymphocyte subset, is decreased [20]. In addition, T-cell response to antigen stimulus is impaired in persons with CKD [21]. CKD patients are also unknown to have the impaired function of neutrophil. In contrast to the decreased count in lymphocytes, the number of neutrophils remain unchanged in ESRD patients [22]. However, as compared to healthy subjects, patients with ESRD seem to have a lower capacity of phagocytosis and greater rate of apoptosis [23, 24].

### Potential mechanisms impairing immune system in CKD

**Fig. 2:**
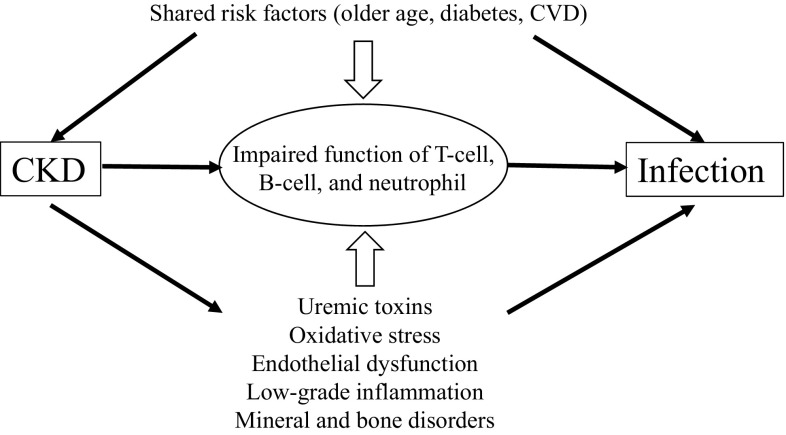
Potential mechanisms increasing infection in chronic kidney disease. *CKD* chronic kidney disease, *CVD* cardiovascular disease

Underlying mechanisms of impaired immune system in CKD are considered multifactorial (Fig. 2**)**. First, shared risk factors of CKD and infection are likely to play an important role. For example, CKD primarily affects older adults, a population at high risk of infection. Immunological changes similar to patients with CKD are also observed in older adults, including reduced lymphocyte production, impaired leukocyte, and neutrophil functions [25, 26]. In addition, diabetes and cardiovascular disease are prevalent among CKD patients, and are known to increase the risk of infection in this population [27–29].

Also, several uremic toxins may contribute to the impaired immune system in CKD. For example, indoxyl sulfate and p-cresyl sulfate are metabolites of tryptophan and tyrosine [30], and previous in vitro studies suggest that these metabolites could impair the leucocyte and endothelial function [30–33]. A couple of small studies of hemodialysis patients suggested the positive association of p-cresol sulfate levels with risk of infection [34, 35]. Another potential metabolite would be trimethylamine-N-oxide (TMAO). TMAO is an oxidative product of trimethylamine [36], and some studies have shown pro-inflammatory aspects of TMAO through the activation of macrophage in patients with CKD, which may ultimately interfere with the immune system [37, 38]. Nonetheless, future studies are still needed to better understand the involvement of these uremic toxins in the impaired immune system in CKD.

Reactive oxygen species (ROS) are important components of the immune response such as activating inflammatory signals and eliminating damaged cells [39]. However, due to their cytotoxicity, excess levels of ROS can actually impair immune function [40]. Among CKD patients, oxidative stress is increased and antioxidant capacity is decreased, and several small studies suggested the link between oxidative stress and impaired immune response [41–43]. However, it is yet to be determined to what extent increased levels of ROS actually contribute to increased risk of infection in CKD patients.

Endothelial cells play an important role in the immune regulation such as cell migration, neutrophil adhesion, and permeability to circulating leukocytes. Several studies have reported the potential link between endothelial dysfunction and impaired immune function [44–46]. Patients with CKD have higher levels of markers for endothelial dysfunction (e.g., soluble P-selectin) compared to healthy controls [47]. Another study reported the association between decreased layer of endothelial surface, known as glycocalyx, and the incidence of albuminuria [48]. Thus, endothelial dysfunction may be another contributing factor for impaired immune response in CKD, and may be relevant to the underlying pathophysiology for elevated infection risk seen in individuals with albuminuria [49]. However, future studies are needed to specifically evaluate whether a measure of endothelial dysfunction, such as flow-mediated dilation [50, 51], is related to the increased risk of infection.

Previous cross-sectional studies showed increased levels of inflammatory cytokines among patients with CKD [52, 53]. For example, in the Chronic Renal Insufficiency Cohort study (eGFR 20–70 ml/min/1.73 m^2^), there was an inverse relationship between plasma levels of inflammatory markers (interleukin-1b, interluekin-1RA, interleukin-6 [IL-6], tumor necrosis factor-alpha [TNF-α], and C-reactive protein [CRP]) and eGFR [53]. Several prospective studies have also shown that an elevation of inflammatory markers such as CRP, IL-6, TNF-α, was associated with increased risk of infection [54–56]. These findings suggest a potential contribution of inflammation to infection, although causality has not yet be determined.

Some evidence suggests that dysregulation of bone and mineral metabolism contributes to the increased risk of infection. Animal studies have suggested that elevated serum level of fibroblast growth factor 23 (FGF23) disrupts the leukocyte and innate immune function [57, 58]. Among patients on dialysis in the Hemodialysis (HEMO) Study, patients in the highest quartile for 25-hydroxyvitamin D had a 33% lower risk of infectious events compared to the lowest quartile; whereas those in the highest quartile FGF23 had a 57% higher risk compared to the lowest quartile [59]. Similar results were also observed for elderly adults [60], as well as in the general population [61]. However, whether mineral and bone disorders can be targeted for an intervention to reduce infection risk is unknown, although a recent meta-analysis reported the protective effects of vitamin D supplementation on reducing respiratory infection in the general population [62].

## Prevention strategies

Some types of infection are preventable through vaccinations such as the influenza and pneumococcal vaccine. Thus, adherence to vaccine recommendations should be the central strategy for reducing risk of vaccine-preventable infections [63, 64]. In addition, there are several non-vaccine prevention measures (e.g., standard preventative measures for hospital-acquired infection), which are also applicable to individuals with CKD.

### Vaccination

Influenza vaccination is probably the most feasible and effective strategy to reduce influenza-related diseases [63, 64]. Although influenza vaccination is beneficial to all age groups, it is particularly important for those at high risk (e.g., older adults, individuals with chronic conditions). In the 2013 KDIGO guideline, annual vaccination with influenza vaccine is recommended to all adults with CKD unless contraindicated [19].

Previous studies in the general population have consistently shown protective effects of influenza vaccination in reducing risk of influenza-related complications by 20–40% [65–67]. However, the effectiveness is less clear among patients with CKD. In ESRD populations in the US, influenza vaccination was non-significantly associated with 10–15% lower risks of hospitalization with influenza/pneumonia [68, 69]. Similarly, a Taiwanese study of hemodialysis patients reported that the receipt of influenza vaccination was associated with ~ 20% lower risks of hospitalization with pneumonia/influenza [70]. These findings suggest that influenza vaccine may be less effective in patients with advanced CKD compared to the general population.

Reduced effectiveness of influenza vaccine in advanced CKD may be due to poorer antibody response to influenza vaccination compared to non-CKD [71–76]. Chang et al. studied antibody response to a single dose H1N1/09 vaccine among 110 hemodialysis patients and 173 healthy controls, and found that the seroconversion rate was 24.5% among hemodialysis patients compared to 86.7% among healthy controls [71]. However, some studies reported less evident difference between dialysis patients and control groups [76]. Further studies are needed to assess antibody response to influenza vaccination in advanced CKD.

Recent studies showed higher effectiveness of a high-dose or adjuvanted influenza vaccine compared to regular vaccine [77, 78]. However, whether these vaccines could benefit CKD patients is not fully clear. A few studies reported a higher vaccine antibody response with an adjuvanted trivalent influenza vaccine among hemodialysis patients and renal transplant recipients [79, 80]. In contrast, a study assessing one booster influenza vaccination among hemodialysis patients showed no differences in the seroconversion rate between a single dose group and one booster dose group [81]. Taken together, although newer influenza vaccines could theoretically induce a stronger vaccine response, additional studies are needed to assess whether CKD patients would benefit from such vaccines.

Pneumococcal vaccination is also an effective strategy to prevent diseases caused by *Streptococcus Pneumoniae*. Currently, two vaccine types, pneumococcal conjugate vaccine (PCV) and pneumococcal polysaccharide vaccine (PPSV), are available. In the 2013 KDIGO guideline [19], pneumococcal vaccination is recommended to all adults with eGFR < 30 ml/min/1.73 m^2^ and those at high risk of pneumococcal infection, such as individuals with nephrotic syndrome, diabetes, or those on immunosuppressive drugs. In addition, revaccination is recommended for adults with CKD within 5 years after receiving pneumococcal vaccination [19].

In a landmark trial of the Community-Acquired Pneumonia Immunization Trial in Adults (CAPITA), PCV13 reduced the risk of community-acquired pneumonia due to vaccine-type strains by 46% in community-dwelling adults aged 65 years or older [82]. PCV13 could induce a stronger vaccine response than PPSV23, but the effectiveness is considered comparable [83]. Whether these data may be generalizable to individuals with CKD is unknown, but some observational studies suggested an improved survival among dialysis patients with pneumococcal vaccination compared to those without [84, 85].

To maintain the adequate immunogenicity, some experts suggest a booster dose of pneumococcal vaccination for patients with CKD [86], since individuals with CKD may have a faster decline in the antibody titers post-vaccination [87–91]. However, its benefits have been controversial. Tobudic et al. reported that the prime-boost strategy did not result in the increased antibody response among transplant patients [92]. Other previous studies have been limited by small number of study subjects and only investigating patients with ESRD. Thus, future studies are needed to assess the effectiveness of pneumococcal vaccination in a broader range of CKD and determine the optimal dose and vaccine delivery strategy.

### Other strategies

Besides vaccine programs, there are several general approaches for preventing infection, which are also applicable to individuals with CKD. Patients with CKD have a high risk of all-cause hospitalization [93], and thus prevention of hospital acquired infections is crucial [94]. Medical devices such as ventilator, central venous catheter, and urinary catheter are frequently used for CKD patients, and are important sources of infection. Standard preventative measures such as good hand hygiene, maximal barrier precautions during the procedure, and prompt removal of devices are critical to minimize the chance of device associated infections [95]. In addition, some active interventions such as quality-improvement interventions [96] and clinical decision support systems (e.g., reminders for preventive care) [97] are shown to be effective but can be expensive. From the perspective of policymaking, these active interventions may be cost-effective when targeted to CKD patients given their high vulnerability to infection. Finally, antibiotic prophylaxis before some invasive procedures such as major surgeries (e.g., cardiac and abdominal surgery) [98] and dental procedures [99] are also generally encouraged to all patients including those with CKD. However, CKD patients may have a high prevalence of multidrug-resistant organism colonization [100], which may complicate the clinical management concerning antibiotic prophylaxis in this clinical population.

## Future research directions

Despite the advancement in the management of CKD, there remains a substantial knowledge gap in the epidemiology of infectious disease in CKD. Future studies should characterize the incidence of overall and cause-specific infection across the spectrum of CKD, particularly including albuminuria stages. Additionally, whether these infections affect the subsequent outcomes of CKD, and if so, to what extent, should be assessed. In addition, we should better understand mechanisms elevating the risk of infection in CKD patients, which would have implications on preventive and therapeutic strategies for infection in CKD. Finally, effective strategies to maximize the benefits of vaccination programs should be developed to prevent vaccine-preventable diseases and improve the outcomes among patients with CKD.

## Conclusions

A body of evidence demonstrated a high risk of infection even at mild to moderate stages of CKD. Nonetheless, infection has been underrecognized and understudied as a complication of CKD. Although several recent studies reported the increased risk of infection among individuals with reduced GFR, definitions of infection varied across studies, and statistical powers were limited in persons with eGFR < 30 ml/min/1.73 m^2^ not requiring renal replacement therapy. Thus, the actual burden of overall and type-specific infection across CKD stages is yet to be determined. Additionally, more studies are needed to quantify the burden of infection associated with albuminuria. Incomplete understanding of underlying mechanisms may preclude us from considering and planning effective preventive strategies for infection in CKD patients. As true in the entire population, vaccination is a major prevention approach for some types of infection in CKD populations, but whether its uptake is optimal is unknown and a few studies raise a question regarding the effectiveness of regular vaccinations in CKD patients. As the number of individuals with CKD is growing globally, it is time to focus on infectious disease as a complication of CKD and advance our understandings to reduce the burden of infection in CKD.
