# Correction to: Clinical epidemiology of infectious disease among patients with chronic kidney disease

**DOI:** 10.1007/s10157-019-01708-7

**Published:** 2019-03-02

**Authors:** Junichi Ishigami, Kunihiro Matsushita

**Affiliations:** Department of Epidemiology, Welch Center for Prevention, Epidemiology, and Clinical Research, Johns Hopkins Bloomberg School of Public Health, 2024 E. Monument St., Suite 2-600, Baltimore, MD 21287 USA

## Correction to: Clinical and Experimental Nephrology 10.1007/s10157-018-1641-8

The article “Clinical epidemiology of infectious disease among patients with chronic kidney disease”, written by Junichi Ishigami and Kunihiro Matsushita, was originally published electronically on the publisher’s internet portal (currently SpringerLink) on 3rd September 2018 without open access. With the author(s)’ decision to opt for Open Choice the copyright of the article changed on 19th February 2019 to © The Author(s) 2019 and the article is forthwith distributed under the terms of the Creative Commons Attribution 4.0 International License (http://creativecommons.org/licenses/by/4.0/),which permits use, duplication, adaptation, distribution and reproduction in any medium or format, as long as you give appropriate credit to the original author(s) and the source, provide a link to the Creative Commons license and indicate if changes were made.

The original article has been corrected.

